# ABC-F Proteins Mediate Antibiotic Resistance through Ribosomal Protection

**DOI:** 10.1128/mBio.01975-15

**Published:** 2016-03-22

**Authors:** Liam K. R. Sharkey, Thomas A. Edwards, Alex J. O’Neill

**Affiliations:** Antimicrobial Research Centre, Astbury Centre for Structural Molecular Biology and School of Molecular and Cellular Biology, University of Leeds, Leeds, United Kingdom

## Abstract

Members of the ABC-F subfamily of ATP-binding cassette proteins mediate resistance to a broad array of clinically important antibiotic classes that target the ribosome of Gram-positive pathogens. The mechanism by which these proteins act has been a subject of long-standing controversy, with two competing hypotheses each having gained considerable support: antibiotic efflux versus ribosomal protection. Here, we report on studies employing a combination of bacteriological and biochemical techniques to unravel the mechanism of resistance of these proteins, and provide several lines of evidence that together offer clear support to the ribosomal protection hypothesis. Of particular note, we show that addition of purified ABC-F proteins to an *in vitro* translation assay prompts dose-dependent rescue of translation, and demonstrate that such proteins are capable of displacing antibiotic from the ribosome *in vitro*. To our knowledge, these experiments constitute the first direct evidence that ABC-F proteins mediate antibiotic resistance through ribosomal protection.

## IMPORTANCE

Antimicrobial resistance ranks among the greatest threats currently facing human health. Elucidation of the mechanisms by which microorganisms resist the effect of antibiotics is central to understanding the biology of this phenomenon and has the potential to inform the development of new drugs capable of blocking or circumventing resistance. Members of the ABC-F family, which include *lsa*(A), *msr*(A), *optr*(A), and *vga*(A), collectively yield resistance to a broader range of clinically significant antibiotic classes than any other family of resistance determinants, although their mechanism of action has been controversial since their discovery 25 years ago. Here we present the first direct evidence that proteins of the ABC-F family act to protect the bacterial ribosome from antibiotic-mediated inhibition.

## INTRODUCTION

Antibiotic resistance undermines effective antibacterial chemotherapy and represents a major threat to global public health (1, 2). A comprehensive response to this problem includes gaining a detailed understanding of the mechanisms by which antibiotic resistance is mediated, particularly since such information could inform the development of novel antibacterial agents able to overcome or circumvent extant resistance phenotypes. While the majority of clinically important antibiotic resistance mechanisms are by now well characterized (3), some key gaps in our knowledge remain. One such gap concerns the mechanism by which ABC-F proteins mediate resistance to a broad array of clinically important antibiotic classes that target protein synthesis in Gram-positive pathogens.

**FIG 1:**
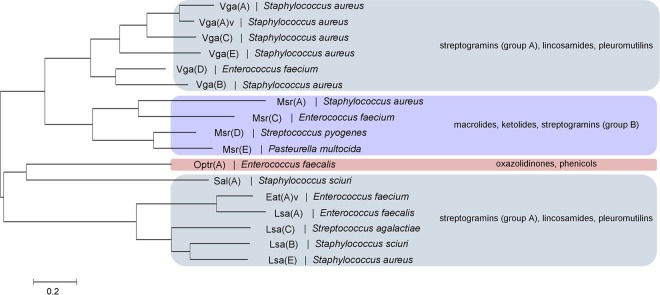
Phylogenetic tree and antibiotic resistance profiles of the ARE ABC-F proteins found in representative Gram-positive pathogens. The tree was generated using the maximum likelihood method with the MEGA 6.0.6 software package (47). An overview of the antibiotic resistance phenotypes conferred by the different subgroups of determinant is given at the right of the figure, denoted by colored boxes (although variations in individual resistance phenotypes within each subgroup are not shown).

ABC-F proteins are found across all three domains of life, and comprise a single polypeptide containing two ATP-binding cassette (ABC) domains separated by a linker of ~80 amino acids. In contrast to canonical ABC transporters, the ABC portions of ABC-F proteins are not fused to transmembrane domains (TMDs), nor are they genetically associated with TMDs in operons (4). While it may be that some ABC-F proteins associate with TMDs to mediate transport across membranes, it is nevertheless apparent that members of this family participate in biological processes other than transport, including DNA repair, enzyme regulation, and translational control (5). In Gram-positive bacteria, a subgroup of the ABC-F proteins mediates resistance to antibiotics that exert their action on the ribosome. These proteins, referred to here as antibiotic resistance (ARE) ABC-F proteins, are found in both antibiotic-producing bacteria (e.g., the streptomycetes) and in pathogenic bacteria that include the staphylococci, streptococci, and enterococci (4, 6) (Fig. 1). Collectively, the ARE ABC-F family of proteins mediates resistance to the majority of antibiotic classes that bind to the 50S subunit of the ribosome, including the ketolides (7), lincosamides (8, 9), macrolides (10), oxazolidinones (11), phenicols (11), pleuromutilins (12), and streptogramins of groups A (9, 13) and B (10) (Fig. 1). However, no single ARE ABC-F determinant confers resistance to every listed class, and three phenotypic resistance profiles are distinguished in clinical isolates. Combined lincosamide and streptogramin A (and sometimes pleuromutilin) resistance, referred to as the LS_A_ (or LS_A_P) phenotype, is conferred by *vga-*, *lsa-*, and *sal-*type genes (9, 14, 15), concurrent resistance to macrolides and group B streptogramins (and sometimes ketolides) (MS_B_ phenotype) by the *msr-*type determinants (7, 10), and resistance to phenicols and oxazolidinones by the recently identified *optrA* gene (11) (Fig. 1).

The mechanism by which the ARE ABC-F proteins mediate antibiotic resistance has been a subject of long-standing controversy, with two competing hypotheses having each attracted considerable support (4, 6, 10, 16–20). The efflux hypothesis posits that ARE ABC-F proteins associate with as-yet-unidentified TMDs to form a functional efflux complex capable of exporting antibiotics out of the cell, while the ribosomal protection hypothesis suggests that these resistance proteins act instead to reduce the accessibility or affinity of the antibiotic binding sites on the 50S subunit of the ribosome, thereby directly protecting the translational machinery from antibiotic-mediated inhibition (16). In proposed support of the efflux hypothesis, previous work has reported membrane localization of Vga(A) in *Staphylococcus epidermidis* (18, 21), and evidence has been obtained for interaction between the streptococcal ARE ABC-F protein Msr(D) and the major facilitator protein Mef(E) when both are heterologously expressed in *Escherichia coli* (19). Furthermore, studies showing that staphylococci expressing ARE ABC-F resistance determinants exhibit decreased accumulation of antibiotic classes that fall within their phenotypic resistance profile (8, 10, 16, 22, 23) have been considered evidence of efflux. However, subsequent work has refuted this interpretation, demonstrating that such uptake studies are incapable of distinguishing drug efflux from ribosomal protection as decreased accumulation of antibiotics will also result from protection of ribosomes (16), which ordinarily act as an intracellular “sink” to increase drug accumulation (24).

Direct evidence for the ribosomal protection hypothesis is similarly lacking. However, the specificity of the ARE ABC-F resistance mechanism for multiple, structurally unrelated classes of protein synthesis inhibitors is easier to interpret in the context of ribosomal protection, and several ABC proteins not involved in antibiotic resistance have recently been shown to interact directly with the ribosome or with ribosomally associated proteins (4, 25).

In order to clarify the mechanism of the ARE ABC-F proteins, we have studied the action of members of this family using bacteriological and biochemical assays, and thereby provide the first direct evidence for a resistance mechanism involving ribosomal protection.

## RESULTS

### The antibiotic resistance phenotype conferred by ARE ABC-F proteins is suggestive of ribosomal protection.

In initial experiments using staphylococci expressing *vga*(A), we sought preliminary support for a mechanism of resistance involving either ribosomal protection or efflux. Previous studies have noted a correlation between the resistance phenotypes mediated by ARE ABC-F proteins and the extent of overlap between binding sites of protein synthesis inhibitors within the PTC and peptide exit tunnel of the 50S subunit (16, 17). A testable prediction is that, if Vga(A) does indeed mediate resistance through ribosomal protection, this protein would likely offer cross-resistance to further classes of structurally unrelated antibiotic that bind the ribosome in close proximity to the group A streptogramins. To examine this, we performed susceptibility determinations with *Staphylococcus aureus* RN4220(pSEPSA5:*vga*(A)) using a panel of 50S-targeted antibiotics (see Table S1 in the supplemental material). In line with published data, expression of *vga*(A) conferred reduced susceptibility to virginiamycin M (64-fold), lincomycin (8-fold), and retapamulin (4-fold) but had no impact on susceptibility to erythromycin, florfenicol, or linezolid. However, we also detected a 4-fold decrease in susceptibility to the 16-member macrolides carbomycin A and leucomycin A1, structurally related antibiotics whose binding site on the ribosome is predicted to partially overlap that of members of the group A streptogramins (26). To our knowledge, this is the first report of a *vga*-type ARE ABC-F protein mediating any degree of macrolide resistance, and offers further indirect support for a mechanism of resistance involving ribosomal protection.

A common feature of antibiotic resistance mechanisms involving efflux is that, when coresident in a bacterial cell with a determinant conferring resistance to the same antibacterial agent through protection of the drug target, a synergistic or additive increase in resistance is observed. For example, *S. aureus* strains expressing both the tetracycline ribosome protection protein (RPP), Tet(M), and the tetracycline efflux pump, Tet(K), exhibit a substantial enhancement of tetracycline resistance compared with strains expressing only one of these resistance mechanisms (27). In contrast, no such enhancement in antibiotic resistance may be observed when two resistance determinants, both of which act at the level of the drug target, coexist in a bacterial cell. For example, in fusidic acid-resistant strains of *S. aureus* carrying both resistance polymorphisms in the drug target (EF-G) and a horizontally acquired fusidic acid resistance gene (*fusB*), the level of resistance observed does not exceed that of the determinant that alone provides the greatest degree of resistance (28). We therefore reasoned that failure of Vga(A) to exhibit an additive or synergistic effect when coresident in *S. aureus* with a ribosomal protection mechanism of streptogramin resistance would suggest that Vga(A) does not mediate resistance by efflux. To test this, we created a strain carrying both *vga*(A) and *cfr*, the latter of which encodes an rRNA methyltransferase capable of methylating 23S rRNA at position 8 of adenine 2503 (*E. coli* numbering) and thereby protects ribosomes from inhibition by several antibiotic classes, including those encompassed within the spectrum of resistance of Vga(A) (29). Susceptibility to virginiamycin M, lincomycin, and linezolid was determined for *S. aureus* RN4220 expressing Vga(A) alone or Vga(A) and Cfr. As expected, expression of Vga(A) alone mediated resistance to virginiamycin M and lincomycin, but not to linezolid, while expression of Cfr alone gave resistance to all three drugs (see Table S2 in the supplemental material). Coexpression of both resistance determinants did not confer a further decrease in susceptibility to any of the drugs beyond that exhibited by the strain solely expressing Cfr, establishing that coexpression of the two resistance proteins does not produce an additive or synergistic effect (Table S2) and further reinforcing the idea that resistance is more likely mediated through ribosomal protection than efflux.

### Purified ARE ABC-F proteins mediate specific and dose-dependent protection of staphylococcal translation from antibiotic inhibition *in vitro*.

Since our bacteriological studies pointed toward ribosomal protection as the likely mechanism of resistance of ARE ABC-F proteins, we sought to directly test the ability of these proteins to protect the translation apparatus from antibiotic-mediated inhibition. We therefore overexpressed and purified recombinant Vga(A) from *E. coli* for addition into an *S. aureus* in vitro coupled transcription/translation (T/T) assay. Addition of a C-terminal hexahistidine (6×His) tag to Vga(A) has previously been demonstrated not to perturb the ability of the protein to mediate resistance in whole cells (21), and we therefore expressed Vga(A) fused to this tag to facilitate purification by immobilized-metal affinity chromatography (IMAC). Effective purification of Vga(A) required removal of contaminating nucleic acids (21), which was achieved by the addition of 2 M NaCl during IMAC and subsequent gel filtration purification steps.

**FIG 2:**
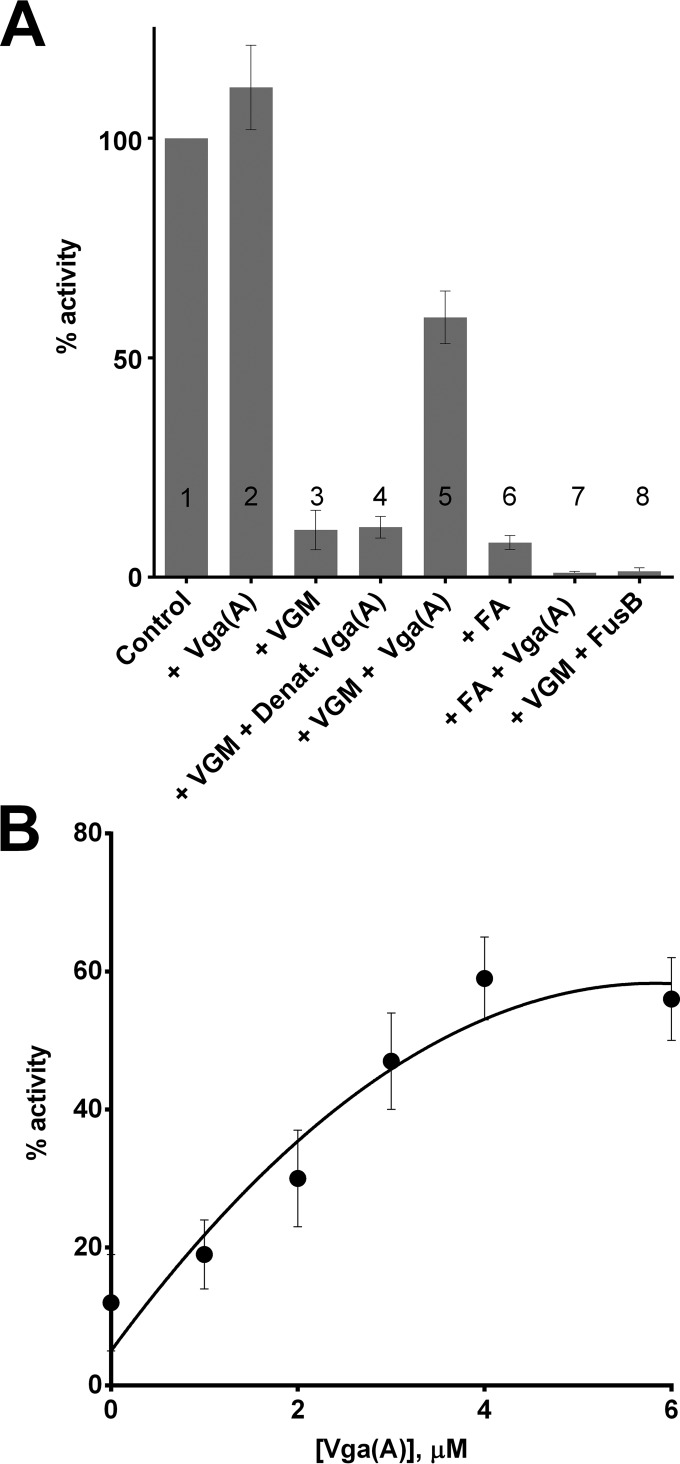
Vga(A) protects an *S. aureus*-derived T/T assay from inhibition by virginiamycin M. (A) Column 1 shows an uninhibited T/T assay with no addition of exogenous protein, whilst column 2 shows an uninhibited assay with the addition of 4 µM Vga(A). In columns 3 and 5, 4 µM Vga(A) added to a T/T assay mixture containing ≥IC_90_ of virginiamycin M (VGM [column 3]) rescued protein synthesis. In columns 3, 4, and 8, addition of 4 µM heat-denatured (Denat.) Vga(A) (column 4) or 4 µM fusidic acid resistance protein FusB (column 8) failed to rescue protein synthesis from inhibition by virginiamycin M (column 3). In columns 6 and 7, addition of 4 µM Vga(A) to a T/T assay mixture containing ≥IC_90_ of fusidic acid (FA) did not rescue protein synthesis. (B) Dose-dependent rescue of protein synthesis by Vga(A) from inhibition with ≥IC_90_ of virginiamycin M. Results are means from at least three independent determinations, and error bars show standard deviations.

Introduction of 4 µM purified Vga(A) into T/T assays inhibited with a ≥90% inhibitory concentration (IC_90_) of virginiamycin M resulted in substantial restoration of translation, with activity rising to ~60% of that of the untreated control (Fig. 2A). In contrast, Vga(A) offered no protection against inhibition of translation by a structurally and mechanistically unrelated inhibitor of protein synthesis (fusidic acid) (Fig. 2A). The ability to rescue translation from virginiamycin M was a specific property of Vga(A), since no protection against this antibiotic was observed when the fusidic acid resistance protein FusB (28) was substituted for Vga(A), and protection was also lost upon heat denaturation of Vga(A) (Fig. 2A). Protection of translation from virginiamycin M by Vga(A) exhibited dose dependence in the concentration range between ~1 and 4 µM Vga(A) (Fig. 2A).

**FIG 3:**
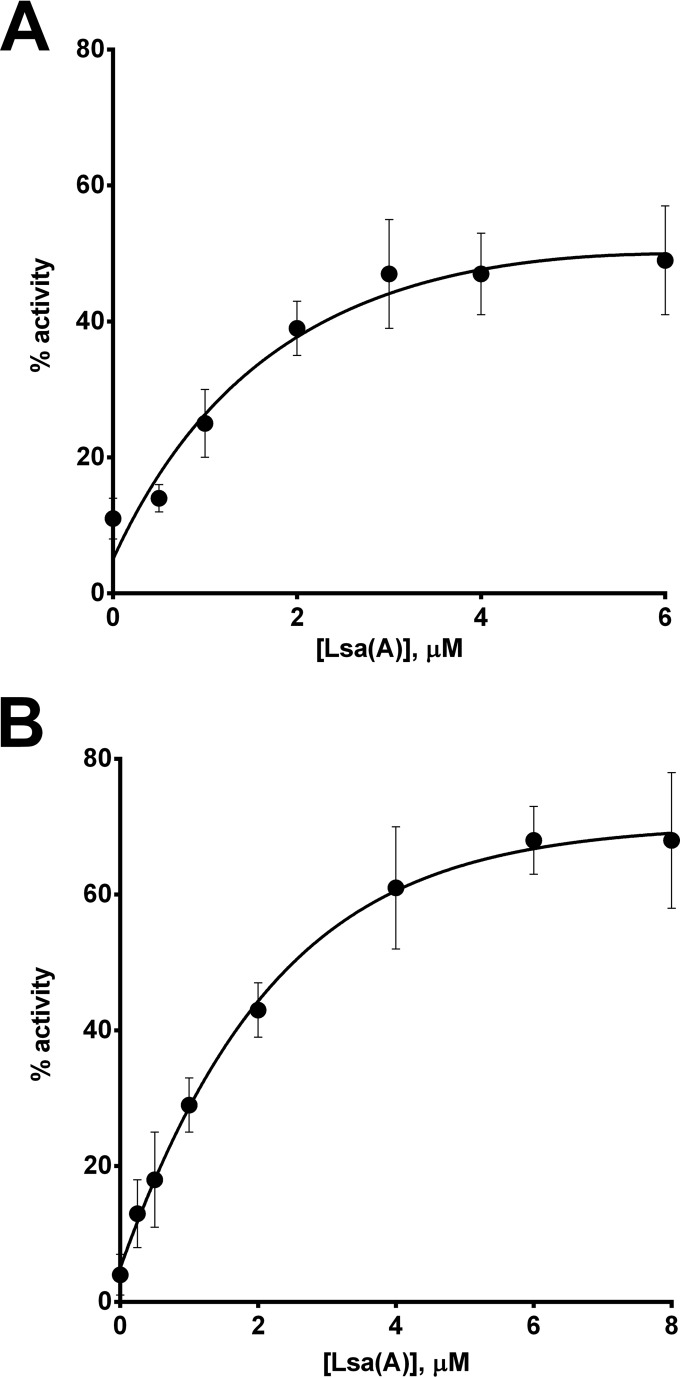
Lsa(A) mediates dose-dependent protection of a *S. aureus*-derived transcription/translation assay from inhibition by virginiamycin M (A) and lincomycin (B). Results are means from at least three independent determinations, and error bars show standard deviations.

To confirm that the ability to protect the translation apparatus from antibiotic-mediated inhibition is not unique to Vga(A) but is a property shared by other ARE ABC-F proteins, we also purified and tested the ability of a phylogenetically distant member of this family, Lsa(A) (Fig. 1), to rescue translation. Lsa(A) was overexpressed in *E. coli* with N-terminal His and SUMO tags to maximize protein solubility. Both tags were cleaved following an initial IMAC purification step, yielding untagged Lsa(A) with an additional N-terminal glycine residue, which was subsequently purified to homogeneity by gel filtration. Purified Lsa(A) was titrated into T/T reaction mixtures inhibited with a ≥IC_90_ of virginiamycin (Fig. 3A) or lincomycin (Fig. 3B). As for Vga(A), rescue of translation was concentration dependent up to ~4 to 6 µM and at the highest protein concentrations tested restored translation activity to at least ~50% in each case (Fig. 3A and B).

### Recapitulation of resistance phenotypes associated with ARE ABC-F proteins in an *in vitro* translation assay.

To provide further confirmation that the observed ability of ARE ABC-F proteins to protect an *in vitro* translation assay from antibiotics reflects the activity of these proteins in whole cells and to further explore the phenomenon of protection, we sought to recapitulate in the T/T assay several phenotypes that have been associated with these proteins in bacteria.

**FIG 4:**
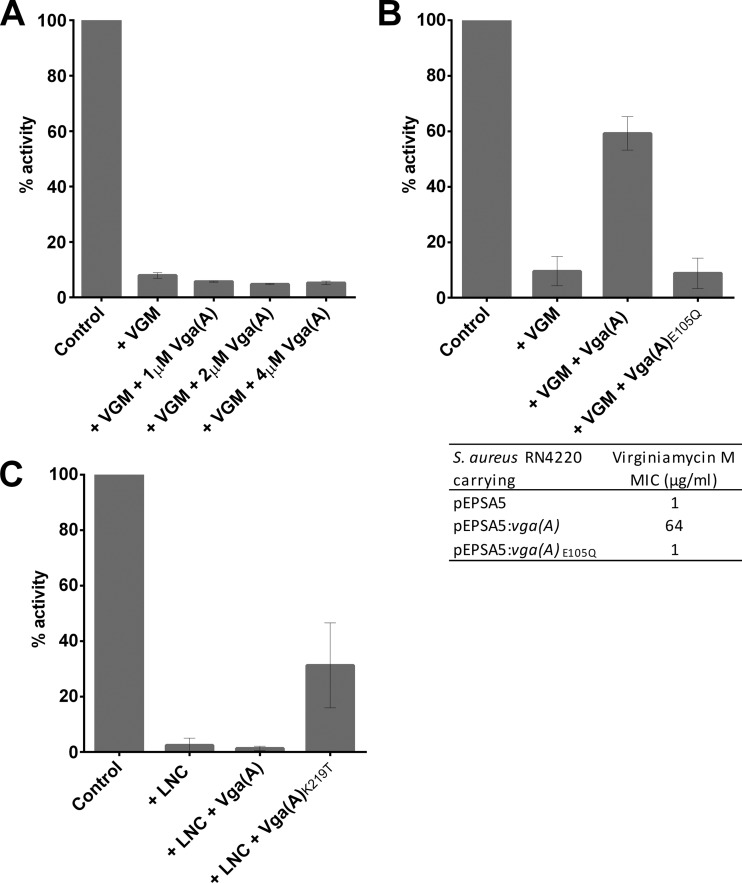
Recapitulation of resistance phenotypes associated with ARE ABC-F proteins *in vitro*. (A) When expressed in *E. coli*, Vga(A) does not confer resistance to virginiamycin M (21); addition of Vga(A) to an *E. coli* T/T assay containing ≥IC_90_ of virginiamycin M (VGM) also failed to restore translational activity. (B) ATPase activity is essential for Vga(A) function (21), and abrogation of ATPase activity of the N-terminal ABC domain rendered Vga(A) inactive when expressed in *S. aureus* RN4220; the purified ATPase-deficient Vga(A)_E105Q_ protein also failed to protect staphylococcal translation from inhibition by virginiamycin M *in vitro*. (C) A single-amino-acid substitution in the interdomain linker expands the resistance spectrum of Vga(A) to encompass lincomycin (20). Addition of the purified Vga(A)_K219T_ to a staphylococcal T/T assay inhibited with a >IC_90_ of lincomycin (LNC) restored translational activity, while addition of the wild-type protein did not. Results are means from at least three independent determinations, and error bars show standard deviations.

The Vga(A) protein is not functional in *E. coli*, failing to confer any reduction in virginiamycin M susceptibility even when detectably overexpressed (21; data not shown). This result was mirrored in an *in vitro* T/T assay using *E. coli* S30 extract; addition of increasing concentrations of Vga(A) (Fig. 4A) and Lsa(A) (data not shown) to maximum of 4 µM into T/T reactions inhibited with ≥IC_90_ of virginiamycin M produced no rescue of translation.

It has previously been demonstrated that substitution for glutamine of the catalytic glutamate residue following the Walker B motif in either nucleotide binding domain of Vga(A) results in a nonfunctional protein incapable of mediating resistance to virginiamycin M in cells of *Staphylococcus epidermidis* (21). We confirmed that this also holds true in cells of *S. aureus*, with expression of Vga(A)_E105Q_ in *S. aureus* RN4220 having no effect on virginiamycin M susceptibility (Fig. 4B). The same loss of the ability of Vga(A)_E105Q_ to mediate virginiamycin M resistance could also be demonstrated *in vitro*, with addition of up to 4 µM purified Vga(A)_E105Q_ to a T/T assay employing *S. aureus* S30 extract producing no restoration of translation activity (Fig. 4B).

A single-amino-acid substitution (K219T) in the linker region between the two nucleotide binding domains of Vga(A) has recently been reported to increase the level of phenotypic resistance to lincosamides from low level (4-fold) to high level (64-fold) (20). This shift in resistance profile was successfully recapitulated in the *S. aureus* T/T assay; addition of purified Vga(A)_K219T_ to a T/T reaction mixture inhibited with lincomycin resulted in restoration of translation activity to ~30% of that of the uninhibited control, while 4 µM wild-type Vga(A) did not detectably protect translation against lincomycin (Fig. 4C).

### Lsa(A) prevents binding of antibiotic to staphylococcal ribosomes, and displaces ribosome-bound antibiotic.

**FIG 5:**
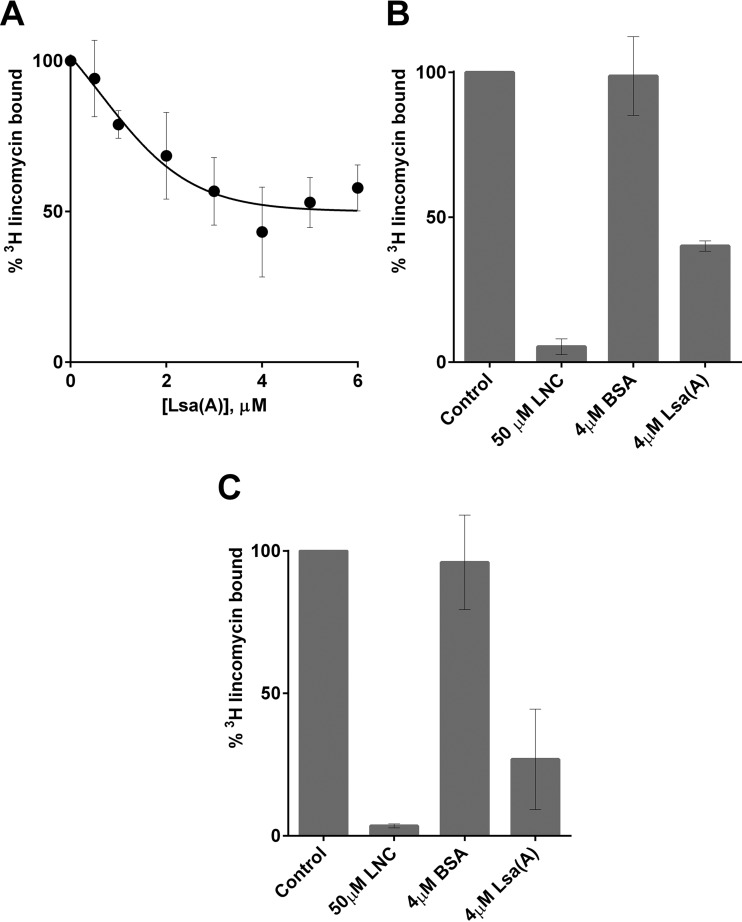
Lsa(A) prevents binding of lincomycin to staphylococcal ribosomes, and displaces ribosome-bound lincomycin. (A) Preincubation of increasing concentrations of Lsa(A) with 0.5 µM staphylococcal ribosomes caused a reduction in binding of [^3^H]lincomycin. (B) Preincubation of 0.5 µM ribosomes with a 50× excess of unlabeled lincomycin (LNC) decreased subsequent binding by [^3^H]lincomycin (column 2 versus 1). Preincubation with 4 µM BSA did not protect ribosomes from binding by [^3^H]lincomycin (columns 3 and 1). Addition of 4 µM Lsa(A) resulted in decreased association of [^3^H]lincomycin with ribosomes (column 4 versus 1). (C) Addition of a 50× excess of unlabeled lincomycin caused dissociation of [^3^H]lincomycin prebound to staphylococcal ribosomes (column 2 versus 1), as did addition of 4 µM Lsa(A) (column 4 versus 1); however, addition of BSA did not (column 3 versus 1). Results are means from at least three independent determinations, and error bars show standard deviations.

We sought to examine whether the ARE ABC-F proteins protect the translation apparatus from antibiotic-mediated inhibition by directly interfering with the interaction between the antibiotic and the ribosome. In the first instance, we evaluated the ability of the Lsa(A) protein to prevent binding of ^3^H-radiolabeled lincomycin to purified staphylococcal ribosomes. Preincubation of between 1:1 and 8:1 molar ratios of Lsa(A) to ribosomes prior to the addition of [^3^H]lincomycin resulted in a dose-dependent decrease in subsequent binding of lincomycin to ribosomes, before reaching a plateau past which addition of Lsa(A) caused no further reduction in lincomycin binding (Fig. 5A). In contrast, preincubation of ribosomes with an 8-fold molar excess of an unrelated control protein (bovine serum albumin [BSA]) did not reduce the level of ribosomally associated [^3^H]lincomycin (Fig. 5B).

Subsequently, we examined the ability of Lsa(A) to displace prebound [^3^H]lincomycin from ribosomes. We first confirmed that this assay could demonstrate displacement of radiolabeled lincomycin following addition of a 50-fold excess of the unlabeled drug and that addition of an 8-fold molar excess of BSA had essentially no effect on the level of ribosome-bound drug (Fig. 5C). Addition of an 8-fold molar-excess of Lsa(A) to ribosomes preincubated with a 2-fold molar-excess of drug resulted in a substantial (~73%) reduction in ribosome-associated [^3^H]lincomycin (Fig. 5C).

## DISCUSSION

The ARE ABC-F proteins collectively yield resistance to a broader range of clinically significant antibiotic classes than any other family of resistance determinants. Despite their importance, the mechanism by which they mediate antibiotic resistance has remained obscure since their discovery 25 years ago (10). Here we have provided several lines of indirect and direct evidence that together reveal a mechanism of resistance involving ribosomal protection. The demonstration that more than one member of this family protects the staphylococcal translation apparatus from antibiotic-mediated inhibition *in vitro*, under conditions where transport cannot occur, coupled with the lack of evidence for drug efflux by the ARE ABC-F proteins, would appear to render the efflux hypothesis redundant.

ARE ABC-F proteins rescue translation from antibiotic-mediated inhibition by driving dissociation of bound antibiotic molecules from the ribosome. The fact that a given ARE ABC-F protein is capable of triggering dissociation of multiple structurally distinct classes of antibiotic, provided these compounds share overlapping binding sites, argues for a simple mechanism of resistance based upon protein-mediated drug displacement. Such a displacement mechanism underlies the only other clinically important example of antibiotic resistance involving ribosomal protection: resistance to tetracyclines mediated by RPPs. These proteins, such as Tet(O) and Tet(M), reach into the tetracycline binding site to directly dislodge the drug (30, 31).

While molecular detail regarding the interaction occurring between ARE ABC-F proteins and the ribosome will be contingent on structural characterization of the complex they form, the recent functional and structural characterization of a non-ARE, but ribosome-binding, ABC-F protein offers a basis for informed speculation. Energy-dependent translational throttle A (EttA) is a bacterial ABC-F protein that binds the ribosome to regulate protein synthesis in response to changing cellular energy levels (32). This protein binds into the E-site of the ribosome, bridging the L1 stalk and P-site, and modulates the conformation of the PTC through contacts with ribosomal proteins, rRNA and P-site fMet-tRNA (33). These interactions occur primarily through the EttA interdomain linker, a well-conserved region of the protein designated the P-site tRNA interaction motif (32). In a recent paper, Lenart et al. (20) noted that this motif is conserved in Vga(A), although extended by an additional 30 amino acids, and demonstrated that single-amino-acid substitutions within this additional region can alter the resistance profile of Vga(A). On this basis, they speculated that the Vga(A) linker may act in an analogous manner to the EttA linker, but with the 30-amino-acid extension allowing further penetration toward the PTC, where it causes dissociation of its target drugs either directly, or through contacts with the P-site tRNA (20). In light of the findings presented here, which collectively confirm that the ARE ABC-F proteins do mediate resistance at the ribosome—and indeed, that these proteins act to displace bound antibiotic from the ribosome—such an explanation for the mechanism of resistance appears compelling. However, it is of note that the interaction of EttA with the ribosomal L1-stalk is mediated by a second functionally important motif, a 44-amino-acid region between the Q-loop and signature motif of the N-terminal ABC domain referred to as the arm region, which is not present in ARE ABC-F proteins. This region is important for determining the specificity of the protein-protein interactions in which other ABC proteins participate (5, 34, 35). Therefore, while the interdomain linker of ARE ABC-F proteins may interact with the P-site tRNA in a similar manner to EttA, the site and mode of binding of the ARE ABC-F proteins to the ribosome may be distinct.

## MATERIALS AND METHODS

### Bacteria and plasmids.

The bacteria and plasmids used or generated in this study are listed in Table 1.

**TABLE 1:** Bacteria and plasmids used in this study

Strain or plasmid	Description	Source or reference
Strains		
*E. faecalis* ATCC 29212	Source of *lsa*(A) gene	ATCC (48)
*S. aureus* RN4220	Restriction-deficient derivative of *S. aureus* 8325-4, used for routine cloning and antibiotic susceptibility testing	49
* E. coli*	**	**
DH5α	For routine cloning procedures	Invitrogen, Paisley, United Kingdom
BL21(λDE3) Gold	For expression of Vga(A)	Agilent Technologies
BL21-CodonPlus(λDE3) RIL	For expression of Lsa(A)	Agilent Technologies
CopyCutter EPI400	To maintain plasmid pSA*luc* at low copy number	Epicenter, Madison, WI
* S. aureus*	**	**
RN4220 *vga*(A)^+^	*S. aureus* RN4220 with *vga*(A) integrated at ϕ11 *attB* locus under control of *cap1a* promoter	This study
RN4220 *vga*(A)^+^(pEPSA5:*cfr*)	*S. aureus* RN4220 *vga*(A)^+^, with *cfr* under control of pT5X promoter on pEPSA5	This study
RN4220(pEPSA5)	RN4220 carrying pEPSA5	This study
RN4220(pEPSA5:*cfr*)	RN4220 carrying *cfr* under control of pT5X promoter on pEPSA5	This study
RN4220(pEPSA5:*vga*(A))	RN4220 carrying *vga*(A) under control of pT5X promoter on pEPSA5	This study
Plasmids		
pEPSA5	*S. aureus*/*E. coli* shuttle vector for expression of genes in *S. aureus* from xylose-inducible promoter pT5X	37
pLL39	Single-copy integration vector for integration at L54a *attB* or ϕ11 *attB* sites on *S. aureus* chromosome	40
pLL2787	Accessory plasmid carrying ϕ11 *int* gene	36
pIVEX2.3d:*vga*(A)	For expression of Vga(A) with C-terminal 6×His tag in *E. coli*	5 Prime GmbH, Düsseldorf, Germany (21, 50)
pBEST	Contains firefly luciferase (*luc*) gene under control of *E. coli tac* promoter	Promega, Madison, WI
pSA*luc*	Modified pBEST plasmid with *luc* gene under control of strong staphylococcal *cap1A* promoter	44
pEPSA5:*vgaA*	For expression of Vga(A) in *S. aureus*	This study
pEPSA5:*vga*_E105Q_	For expression of ATPase-deficient mutant of Vga(A) in *S. aureus*	This study
pEPSA5:*vgaA*_K219T_	For expression of expanded-phenotype mutant of Vga(A)	This study
pEPSA5:*cfr*	For expression of *cfr* in *S. aureus*	This study
pLL39:*vga*(A)	For integration of *vga*(A) onto *S. aureus* chromosome under control of *cap1a* promoter	This study
pIVEX2.3d:*vga*(A)_E105Q_	For expression of ATPase-deficient mutant of Vga(A) with C-terminal 6×His tag in *E. coli*	This study
pIVEX2.3d:*vga*(A)_K219T_	For expression of expanded-phenotype mutant of Vga(A) with C-terminal 6×His tag in *E. coli*	This study
pET28a:SUMO-*lsa*(A)	Modified pET28a expression vector encoding N-terminal 6×His and SUMO (type 3) tags followed by recognition site for U1p protease, used to express Lsa(A)	This study

### Antibiotics, chemicals, and susceptibility testing.

Antibiotics were from Sigma (Poole, United Kingdom), with the exception of tylosin (Cambridge Bioscience, Cambridge, United Kingdom), spiramycin (Cambridge Bioscience), and sparsomycin (E. Cundliffe, University of Leicester). Tritium (^3^H)-labeled lincomycin was synthesized by Quotient Bio Research (Nottingham, United Kingdom).

MICs were determined by exposing bacteria to 2-fold serial dilutions of antibacterial agents in Mueller-Hinton broth 2 (Oxoid, Cambridge, United Kingdom) according to the guidelines provided by the CLSI (36). For MIC determinations using strains carrying antibiotic resistance genes on expression plasmid pEPSA5, 1% (wt/vol) xylose was added to cultures following their dilution to a 0.5 McFarland standard to induce expression from the pT5X promoter.

### DNA manipulation.

The oligonucleotide primers used for PCR are listed in Table S3 in the supplemental material.

For expression in *S. aureus*, PCR amplicons corresponding to *vga*(A) and *lsa*(A), and a synthesized DNA fragment corresponding to *cfr* (GenScript, Piscataway, NJ), were ligated into the staphylococcal expression vector pEPSA5 (37) and introduced into *S. aureus* RN4220 by electroporation (38). In order to assess the activity of Vga(A) in the presence of the *cfr* determinant, a strain of RN4220 was generated carrying *vga*(A) on the chromosome and *cfr* on plasmid pSEPSA5:*cfr.* To achieve this, a PCR amplicon of *vga*(A) downstream of the strong, constitutive promoter *P_cap1A_* (39) was ligated into the integrative vector pLL39 (40) and introduced into *S. aureus* RN4220(pLL2787) (40) by electroporation, whereupon it became integrated into the chromosome at the ϕ11 *attB* site. A construct for overexpression of Vga(A) with a C-terminal 6×His tag in *E. coli* was generated as previously described (21). The *lsa*(A) gene was amplified from *Enterococcus faecalis* ATCC 29212 (NCBI WP_002365053.1) and ligated into a modified pET28b plasmid (Merck KGaA, Darmstadt, Germany) encoding N-terminal 6×His and SUMO tags (41). The resulting expression constructs were transformed into *E. coli* BL21(λDE3) Gold (Agilent Technologies, Cheshire, United Kingdom) and BL21-CodonPlus(λDE3) RIL (Agilent Technologies), respectively. Nucleotide replacements to encode amino acid substitutions E_105_Q and K_219_T in Vga(A) were independently engineered into constructs carrying *vga*(A) using the QuikChange Lightning kit (Agilent Technologies).

### Heterologous expression and purification of proteins.

Vga(A) and Lsa(A) were overexpressed in *E. coli* by autoinduction (42) at 25°C for 4 days, and cells were harvested by centrifugation. Cell pellets were resuspended at 3 ml/g in buffer A (50 mM NaH_2_PO_4_ [pH 8.0], 300 mM NaCl, 10 mM imidazole), incubated with 7,000 U chicken egg-white lysozyme (Sigma), complete EDTA-free protease inhibitor tablets (Roche, Mannheim, Germany), and 17 U Basemuncher endonuclease/ml of suspension (Expedeon, Harston, United Kingdom) for 30 min, and lysed by sonication. Lysates were clarified by centrifugation at 30,000 × *g* for 30 min. Cleared lysate was loaded onto a 25-ml free-flow gravity column (GeneFlow, Elmhurst, United Kingdom) packed with 5 ml Ni-nitrilotriacetic acid (NTA) agarose (Expedeon), washed with buffer A containing 20 mM imidazole and 2 M NaCl, eluted in buffer A containing 250 mM imidazole and 2 M NaCl, and dialyzed overnight into buffer B (20 mM HEPES [pH 7.5], 2 M NaCl, 1 mM dithiothreitol [DTT]). The C-terminal 6×His tag of Vga(A) was not removed, but the N-terminal 6×His and SUMO tags were removed from Lsa(A) by digestion with SUMO protease (Ulp, Thermo, Fisher Scientific, MA) during dialysis. Dialyzed protein was subjected to gel filtration using a Superdex 200 (16/60) column (GE Healthcare, Buckinghamshire, United Kingdom) preequilibrated with buffer B. Lsa(A) was then exchanged into buffer C (50 mM HEPES [pH 7.4]) containing 300 mM NaCl on HiTrap desalting columns (GE Healthcare) and stored at −80°C. Vga(A) was exchanged into buffer C with 50 mM NaCl and further purified using a resource S column by elution with a 500 mM NaCl gradient. Finally, Vga(A) was also exchanged to buffer C containing 300 mM NaCl prior to storage at −80°C. SDS-PAGE and peptide mass fingerprinting were used to detect and confirm the identity of purified proteins, with the latter performed by the University of Leeds Mass Spectrometry Facility. The FusB protein was expressed and purified as described previously (43).

### *In vitro* transcription/translation assays.

Staphylococcal S30 extract was prepared from *S. aureus* RN4220 following the protocol of Murray et al. (44), although the preincubation step was omitted. *E. coli* S30 extract was from Promega (Madison, WI). For T/T assays, an optimized quantity of S30 extract was added into a 25-µl reaction mixture containing 0.1 mM amino acids (Promega), 10 µl S30 premix (Promega), 1 µg of DNA template (pSA*luc* or pBEST*luc* for *S. aureus* and *E. coli* T/T reactions, respectively), and antibiotics and purified protein as required. Reaction mixtures were incubated for 1 h at 37°C, and the level of transcription/translation was quantified by monitoring the expression of luciferase produced from pSA*luc*/pBEST*luc* by the addition of luciferase assay reagent (Promega) and measurement of luminescence.

### Purification of staphylococcal ribosomes and use in antibiotic binding assays.

Ribosomes were purified from S30 fractions of *S. aureus* RN4220 using l-cysteine Sulfolink resin (Thermo Fisher Scientific, Waltham, MA) and ultracentrifugation, as previously described (45). For quantitation, ribosomes were separated on a denaturing agarose gel alongside an RNA standard of known concentration and analyzed by two-dimensional (2D) densitometry using AIDA software (Raytest, Straubenhardt, Germany).

The ability of Lsa(A) to prevent binding of radiolabeled lincomycin to the ribosome was assessed essentially as previously described for tetracycline-ribosome binding studies (46). Ribosomes (500 nM) were preincubated in 50-µl reaction mixtures with Lsa(A), BSA, or unlabeled lincomycin (concentrations described in Results) in assay buffer (10 mM Tris [pH 7.5], 60 mM KCl, 10 mM NH_4_Cl, 300 mM NaCl, 6 mM MgCl_2_, 0.1 mM ATP) at 37°C. After 10 min, 1 µM [^3^H]lincomycin was added, and the reaction mixtures were incubated for a further 10 min before recovering ribosomes on 0.45-µm-pore nitrocellulose filters and two rounds of washing with 200 µl of ice-cold wash buffer (50 mM Tris, 50 mM KCl, 20 mM MgOAc) to remove unbound [^3^H]lincomycin. Ribosome-associated [^3^H]lincomycin was then quantified by scintillation counting.

The ability of Lsa(A) to displace prebound lincomycin from ribosomes was investigated using the same assay conditions; however, ribosomes were preincubated with [^3^H]lincomycin for 10 min at 37°C prior to the addition of Lsa(A), BSA, or unlabeled lincomycin. Reaction mixtures were then incubated for a further 10 min at 37°C, before proceeding to ribosome recovery, washing, and quantitation of ribosome-bound [^3^H]lincomycin as described above.

## SUPPLEMENTAL MATERIAL

Table S1  MICs of 50S-targeted antibiotics against *S. aureus* expressing or lacking Vga(A). Table S1, DOCX file, 0.02 MB

Table S2  MICs of 50S-targeted antibiotics against *S. aureus* RN4220 expressing chromosomally encoded Vga(A) alone, plasmid-encoded Cfr alone, or both together. Table S2, DOCX file, 0.02 MB

Table S3  Oligonucleotide primers used in this study. Restriction sites and sequences complementary to the pLL39 vector used for Gibson assembly are italicized, codons targeted for mutagenesis are underlined, and expression signals (promoters and ribosome binding sites) are shown in boldface. Table S3, DOCX file, 0.02 MB
