# Supplementary material for: ABC-F Proteins Mediate Antibiotic Resistance through Ribosomal Protection
Source: mBio. 2016 Mar 22;7(2):e01975-15. doi: 10.1128/mBio.01975-15 (PMC4807367; doi:10.1128/mBio.01975-15)
Supplement: Table S1 — MICs of 50S-targeted antibiotics against S. aureus expressing or lacking Vga(A). [file mbo001162722st1.docx]

# Supplementary information

**Table S1. MICs of 50S targeted antibiotics against *S. aureus* expressing or lacking Vga(A).**

|  | *S. aureus* RN4220 (pEPSA5) | *S. aureus* RN4220 (pEPSA5:*vga(A)*) |
| --- | --- | --- |
| Blasticidin S | 128 | 128 |
| Carbomycin | 0.5 | 2 |
| Erythromycin | 0.5 | 0.5 |
| Florfenicol | 4 | 4 |
| Leucomycin | 0.25 | 1 |
| Lincomycin | 0.25 | 2 |
| Linezolid | 2 | 2 |
| Puromycin | 8 | 8 |
| Retapamulin | 0.032 | 0.125 |
| Sparsomycin | 32 | 32 |
| Spiramycin | 1 | 1 |
| Tiamulin | 0.25 | 0.25 |
| Tylosin | 0.5 | 0.5 |
| Virginiamycin M | 1 | 64 |
