# Supplementary material for: ABC-F Proteins Mediate Antibiotic Resistance through Ribosomal Protection
Source: mBio. 2016 Mar 22;7(2):e01975-15. doi: 10.1128/mBio.01975-15 (PMC4807367; doi:10.1128/mBio.01975-15)
Supplement: Table S2 — MICs of 50S-targeted antibiotics against S. aureus RN4220 expressing chromosomally encoded Vga(A) alone, plasmid-encoded Cfr alone, or both together. [file mbo001162722st2.docx]

# Supplementary information

**Table S2. MICs of 50S targeted antibiotics against *S. aureus* RN4220 expressing chromosomally-encoded Vga(A) alone, plasmid-encoded Cfr alone, or both together.**

|  | *S. aureus* RN4220 (pEPSA5) | *S. aureus* RN4220 *vga(A)^+^* | *S. aureus* RN4220 (pEPSA5:*cfr*) | *S. aureus* RN4220 *vga(A)^+^* (pEPSA5:*cfr*) |
| --- | --- | --- | --- | --- |
| Lincomycin | 0.25 | 2 | 128 | 128 |
| Linezolid | 2 | 2 | 8 | 8 |
| Virginiamycin M | 1 | 64 | 128 | 128 |
