# Supplementary material for: ABC-F Proteins Mediate Antibiotic Resistance through Ribosomal Protection
Source: mBio. 2016 Mar 22;7(2):e01975-15. doi: 10.1128/mBio.01975-15 (PMC4807367; doi:10.1128/mBio.01975-15)
Supplement: Table S3 — Oligonucleotide primers used in this study. Restriction sites and sequences complementary to the pLL39 vector used for Gibson assembly are italicized, codons targeted for mutagenesis are underlined, and expression signals (promoters and ribosome binding sites) are shown in boldface. [file mbo001162722st3.docx]

# Supplementary information

**Table S3. Oligonucleotide primers used in this study.** Restriction sites and sequences complementary to the pLL39 vector used for Gibson assembly are italicised, codons targeted for mutagenesis are underlined, and expression signals (promoters and ribosome binding sites) are shown in bold.

| Designation | Description | Sequence (5’-3’) |
| --- | --- | --- |
| VgaA_E105Q_fwd | For mutagenesis of catalytic glutamine in N-terminal ABC | aaaaatccagaactgctattagcagatcagccaacaactaacttagataataac |
| VgaA_E105Q_rev | For mutagenesis of catalytic glutamine in N-terminal ABC | gttattatctaagttagttgttggctgatctgctaatagcagttctggattttt |
| VgaA_K219T_fwd | For mutagenesis of the inter-domain linker | aagtatggctttgctcccgttattttgccttcagataaacttaagtttttcg |
| VgaA_K219T_rev | For mutagenesis of the inter-domain linker | cgaaaaacttaagtttatctgaaggcaaaataacgggagcaaagccatactt |
| VgaA_cap1a39_fwd | For introduction of *vga(A)* into plasmid pLL39 under control of the *cap1A* promoter | *agcttagatctaatcgaattcgagctcggtaccc*agagt**ttgcaa**aatatacaggggattata**tataat**ggaaaacaagaaaggaaaat**aggagg**tttatatggcaaaaataatgttaga |
| VgaA_cap1a39_rev | For introduction of *vga(A)* into plasmid pLL39 under control of the *cap1A* promoter | *tgtaggtaataaaaaagcttgcatgcctgcaggtcgactctagaggat*ttatttatccaaatttcttttttca |
| LsaA_28a_fwd | For introduction of *lsa(A)* into pET28a‑SUMO | gcgcta*ggatcc*atgtcgaaaattgaactaaaacaac |
| LsaA_28a_rev | For introduction of *lsa(A)* into pET28a‑SUMO | cgacta*aagctt*ttatgatttcaagacaatttttttatctgt |
| VgaA_pEPSA5_fwd | For introduction of *vga(A)* into pEPSA5 | gcta*gagctc*at**aagagg**atgagaaaatatggcaaaaataatgttagagggact |
| VgaA_pEPSA5_rev | For introduction of *vga(A)* into pEPSA5 | gactc*ggatcc*ttatttatccaaatttcttttttcat |
| Cfr_pEPSA5_fwd | For introduction of *cfr* into pEPSA5 | gcaac*gagctc*at**aggagg**atcagaaaatatgaactttaacaacaaaacgaaatatgg |
| Cfr_pEPSA5_rev | For introduction of *cfr* into pEPSA5 | gcaac*ggatcc*ttactgggagttctgatagttacc |
